# Public Availability of Published Research Data in High-Impact Journals

**DOI:** 10.1371/journal.pone.0024357

**Published:** 2011-09-07

**Authors:** Alawi A. Alsheikh-Ali, Waqas Qureshi, Mouaz H. Al-Mallah, John P. A. Ioannidis

**Affiliations:** 1 Institute of Cardiac Sciences, Sheikh Khalifa Medical City, Abu Dhabi, United Arab Emirates; 2 Department of Medicine, Tufts Clinical and Translational Science Institute, Tufts University School of Medicine, Boston, Massachusetts, United States of America; 3 Department of Medicine, Henry Ford Hospital, Detroit, Michigan, United States of America; 4 King Abdul-Aziz Cardiac Center, National Guard Health Affairs, Riyadh, Kingdom of Saudi Arabia; 5 Wayne State University, Detroit, Michigan, United States of America; 6 Stanford Prevention Research Center, Stanford University School of Medicine, Stanford, California, United States of America; 7 Department of Hygiene and Epidemiology, University of Ioannina School of Medicine, Ioannina, Greece; 8 Biomedical Research Institute, Foundation for Research and Technology-Hellas, Ioannina, Greece; 9 Department of Epidemiology, Harvard School of Public Health, Boston, Massachusetts, United States of America; University Paris Descartes, France

## Abstract

**Background:**

There is increasing interest to make primary data from published research publicly available. We aimed to assess the current status of making research data available in highly-cited journals across the scientific literature.

**Methods and Results:**

We reviewed the first 10 original research papers of 2009 published in the 50 original research journals with the highest impact factor. For each journal we documented the policies related to public availability and sharing of data. Of the 50 journals, 44 (88%) had a statement in their instructions to authors related to public availability and sharing of data. However, there was wide variation in journal requirements, ranging from requiring the sharing of all primary data related to the research to just including a statement in the published manuscript that data can be available on request. Of the 500 assessed papers, 149 (30%) were not subject to any data availability policy. Of the remaining 351 papers that were covered by some data availability policy, 208 papers (59%) did not fully adhere to the data availability instructions of the journals they were published in, most commonly (73%) by not publicly depositing microarray data. The other 143 papers that adhered to the data availability instructions did so by publicly depositing only the specific data type as required, making a statement of willingness to share, or actually sharing all the primary data. Overall, only 47 papers (9%) deposited full primary raw data online. None of the 149 papers not subject to data availability policies made their full primary data publicly available.

**Conclusion:**

A substantial proportion of original research papers published in high-impact journals are either not subject to any data availability policies, or do not adhere to the data availability instructions in their respective journals. This empiric evaluation highlights opportunities for improvement.

## Introduction

The observations of scientists as coded in their primary data constitute a central commodity in the scientific enterprise [1]. Reproduction of research findings and further exploration of related hypotheses require access to these primary data, and their public availability has been a concern for all stakeholders of the scientific process, including regulatory and funding agencies, journal editors, individual researchers, and patients [2], [3], [4], [5], [6], [7]. Recently, efforts have converged to encourage making data, protocols, and analytical codes available, as part of the growing movement of reproducible research [8], [9], [10]. The benefits and challenges of public data availability and data sharing have long been hotly discussed in the scientific community [11], [12], [13]. Recent analyses have empirically highlighted deficiencies in the practice of making primary data and protocols available in peer-reviewed publications[14], [15], [16]. These analyses, however, have focused on either a particular discipline or area of research or were limited to a single journal. To date, there has not been an empiric evaluation of public availability of primary data and related material and protocols across diverse scientific fields or journals. We aimed to assess the current status of these practices in the most highly-cited journals across the scientific literature.

## Methods

We examined the 50 journals with the highest impact factor according to the Journal Citation Reports (Science edition 2007) issued in the Thompson-Institute for Scientific Information Web of Knowledge. Journals that exclusively publish review articles were not included. For each journal, we also reviewed the first 10 original research papers published in 2009.

For each journal we documented the policies related to public availability and sharing of data, where available, and as stated in the instructions to authors on the journal's website (accessed May 2009). This was done by one investigator (AA) and verified by a second (MA). Each paper was reviewed by going through the text, supplementary material and the links available on the online version. We recorded information on country of first and corresponding authors, funding sources, data links and accession numbers, and whether the paper was based on data covered by a journal policy (e.g. paper with microarray data published in a journal requiring public deposition of microarray data). Data extraction from the 500 papers was done by a single investigator (WQ) with cross-checking in the first 50 papers by two investigators (AA and MA). This information was collected in the months of July and August of 2010. Online information was considered missing if links were not available when checking on 2 separate occasions 2 weeks apart. We compared the impact factor of journals where provision of materials and protocols was a condition of publication versus journals with non-binding instructions or no instructions at all using the Kruskall-Wallis analysis of variance test. We compared the proportion of papers depositing full primary data online by status of US government funding and by geographic origin of corresponding author (US versus non-US) using the Chi square test.

## Results

**Figure 1:**
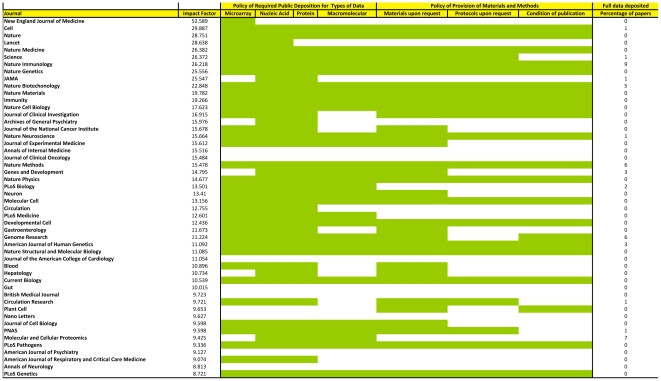
Breakdown of journal policies for public deposition of certain data types, sharing of materials and/or protocols, and whether this is a condition for publication and percentage of papers with fully deposited data.

Of the 50 highest impact factor journals publishing original research, 44 (88%) had a statement in their instructions to authors related to public availability and sharing of data from submitted manuscripts (Appendix S1). However, there was wide variation in journal requirements, ranging from requiring the sharing of all primary data related to the research to just including a statement in the published manuscript that data can be available on request. Some specific types of data had very high frequency of requirement for public deposition. This included public deposition of primary microarray, nucleic acid and protein sequencing data, and macromolecular structures which was required in 36/50 (72%), 40/50 (80%), 39/50 (78%), and 29/50 (58%) journals (Figure 1). Materials and protocols used in published experiments were required to be made available upon request to qualified researchers by 33/50 (66%) and 23/50 (46%) journals, respectively.

When instructing authors on data or material sharing, journals used different phrases to indicate how strict these requirements were. Less than half (22/50, 44%) of the journals explicitly indicated that making materials and/or protocols of the published findings (regardless of method and technology employed) available to other qualified investigators was a condition of publication. In their instructions to authors regarding data sharing, these journals used language such as “with minimal restriction . . . in a timely manner” (e.g. *Cell* family of journals), “non-compliance … may result in denial of future rights to publish” (e.g. *Plant Cell*), or “…a condition of publication . . . is . . to make materials, data and associated protocols promptly available . . . without preconditions” (e.g. *Nature* family of journals). An additional 44% (22/50) of the journals had either a non-binding statement encouraging authors to make their data available (e.g. using language like “it is the responsibility of the authors” or “authors are encouraged to . . .”), or required a data-sharing statement by the authors indicating willingness to share (*Annals of Internal Medicine* and *British Medical Journal*). The remainder 6 journals (12%) had no specific instructions to authors related to data availability. Journals where provision of materials and protocols was a condition of publication had higher impact factors compared to journals with non-binding instructions or no instructions at all (median [25^th^, 75^th^ percentiles]: 15.14 [11.09, 19.78] versus 12.68 [9.72, 15.98] versus 9.83 [9.13, 11.05], P = 0.04 by Kruskal-Wallis analysis of variance).

Of the 500 papers we reviewed (Appendix S2), 149/500 (30%) were not subject to any data availability policy (60 were published in journals without a specific data sharing statement and 89 additional papers contained data not covered by the specific public deposition policy in their journals). Of the remaining 351 papers that were covered by some data availability policy (with or without data deposition as a condition of publication), 208/351 papers (59%) did not fully adhere to the data availability instructions of the journals they were published in, most commonly (73%) by not publicly depositing microarray data. The other 143 papers (of the 351 covered by some data availability policy) that adhered to the data availability instructions of the journals did so by publicly depositing only the specific data type as required, making a statement of willingness to share, or actually sharing all the primary data. Overall, only 47/500 papers (9%) did deposit full primary raw data online; and we were able to verify access in all 47 (sites were accessed in July and August 2010). None of the 149 papers not subject to data availability policies made their full primary data publicly available.

Among papers covered by some data availability policy (n = 351), the proportion depositing full primary data online was not different when US government funding (e.g. National Institutes of Health or National Science Foundation) was listed (15% versus 12% when US funding was not listed, P = 0.42) or the corresponding author was from a US institution (16% versus 11% for non-US corresponding authors, P = 0.20).

## Discussion

The present overview of highly cited journals highlights three main features of the current status of data availability practices in this high impact scientific literature. First, there are heterogeneous instructions to investigators publishing in high impact journals, with some journals requiring public data availability as a condition for publication, others encouraging data sharing but having no binding instructions, and a few journals having no specific instructions at all. Second, nearly a third of the examined sample of 500 papers were not subject to any data availability policies, either because they were published in journals without such policies or with specific policies that do not cover the primary data upon which the research was based. Third, even when research is published in journals with specific instructions regarding data availability, more than half of publications did not adhere to the data availability instructions in their respective journals.

Our findings present a snapshot of data availability practices in recent literature. While the papers we reviewed were from 2009, it is unlikely that the situation has changed much over the past year, and we therefore believe that the present findings represent reasonably well the current state of the literature. Moreover, since the papers we reviewed were likely submitted 6-12 months prior to our recording of journal policies, some journals may have adopted data sharing policies in the interim, hence inflating our estimate of lack of adherence to data sharing policies. However, it is doubtful that policies related to data sharing have changed substantially over such a short period of time. We also focused our analysis on high impact journals, since the research that they publish has a pivotal role in the evolution of scientific investigation and it is essential that this pivotal research is reproducible. It is not likely that data availability practices are more common and more efficient in other journals with lower impact factor - the opposite seems more plausible, if anything. Therefore the present findings may well overestimate the prevalence of effective data sharing among investigators publishing across all peer-reviewed journals. In fact, some types of biomedical studies, in particular traditional epidemiological/observational investigations, may be underrepresented in our sample as compared with molecular and other clinical research. Some of these types of underrepresented studies have no established history of public data repositories and thus primary data availability may be a more critical deficiency in these fields. It is also worth noting that the association between higher impact factor and conditioning publication upon provision of materials/protocols may be confounded by type of journal, as experimental/basic science journals that typically have such conditions tend to have higher impact factors.

While this analysis highlights an important element of data sharing, that of public availability of primary data, there are other elements not evaluated here but still important to make the data sharing culture functional and efficient. For example, a statement of willingness to share raw data by the primary investigators does not always translate into true availability of data when requested by independent scientists [15]. Empirical studies suggest that data withholding is not uncommon in the scientific community and may be influenced by industry relationships, perceptions of proprietary information and scientific priority, lack of resources, and personal investigator training and stances towards data sharing[17], [18], [19]. Moreover, while all data web links of full primary datasets were verified as functioning in our analysis, this may reflect the temporal proximity of our analysis to the publication date of the articles, and some of these links may become unavailable a few years later [20].

Legislation to make results of clinical trials publicly available within one year of study completion may promote the culture of transparency in clinical trials research, but at present such legislation does not mandate making raw data from clinical trials publicly available [21]. Indeed, widespread availability of clinical trial data may be hampered by financial incentives of journals to publish industry-sponsored trials, many of which may be bound by confidentiality agreements [22], [23], [24]. Data sharing may be enhanced when granting agencies require investigators to share data but regulatory barriers remain [25], [26].

Finally, for data that was made available by investigators, we did not attempt to replicate their findings. Even when data are publicly available, published results are often not reproducible by independent investigators due to incomplete annotation or specification of data processing and analyses [14].

This empiric evaluation highlights opportunities for improvement. Journals should adopt more routinely policies for data sharing, expanding the types of data that are subject to public sharing policies with the ultimate target of covering all types of data. Moreover, it is essential to develop mechanisms for journals to ensure that existing data availability policies are consistently followed by researchers and published research findings are easily reproducible.

## Supporting Information

Appendix S1 **Summary of journal policies as they relate to data sharing.**
(XLS)

Appendix S2 **Summary of extracted data from the 500 papers reviewed for the present analysis.**
(XLS)
